# Machine Learning Electronic Health Record Identification of Patients with Rheumatoid Arthritis: Algorithm Pipeline Development and Validation Study

**DOI:** 10.2196/23930

**Published:** 2020-11-30

**Authors:** Tjardo D Maarseveen, Timo Meinderink, Marcel J T Reinders, Johannes Knitza, Tom W J Huizinga, Arnd Kleyer, David Simon, Erik B van den Akker, Rachel Knevel

**Affiliations:** 1 Department of Rheumatology Leiden University Medical Center Leiden Netherlands; 2 Department of Internal Medicine 3 Friedrich-Alexander University Erlangen‐Nuremberg Erlangen Germany; 3 Deutsches Zentrum für Immuntherapie Erlangen-Nuremberg and Universitätsklinikum Erlangen Germany; 4 Leiden Computational Biology Centre Leiden University Medical Center Leiden Netherlands; 5 Molecular Epidemiology Leiden University Medical Center Leiden Netherlands; 6 Division of Rheumatology, Inflammation and Immunity Brigham and Women's Hospital Harvard Medical School Boston, MA United States

**Keywords:** Supervised machine learning, Electronic Health Records, Natural Language Processing, Support Vector Machine, Gradient Boosting, Rheumatoid Arthritis

## Abstract

**Background:**

Financial codes are often used to extract diagnoses from electronic health records. This approach is prone to false positives. Alternatively, queries are constructed, but these are highly center and language specific. A tantalizing alternative is the automatic identification of patients by employing machine learning on format-free text entries.

**Objective:**

The aim of this study was to develop an easily implementable workflow that builds a machine learning algorithm capable of accurately identifying patients with rheumatoid arthritis from format-free text fields in electronic health records.

**Methods:**

Two electronic health record data sets were employed: Leiden (n=3000) and Erlangen (n=4771). Using a portion of the Leiden data (n=2000), we compared 6 different machine learning methods and a naïve word-matching algorithm using 10-fold cross-validation. Performances were compared using the area under the receiver operating characteristic curve (AUROC) and the area under the precision recall curve (AUPRC), and F1 score was used as the primary criterion for selecting the best method to build a classifying algorithm. We selected the optimal threshold of positive predictive value for case identification based on the output of the best method in the training data. This validation workflow was subsequently applied to a portion of the Erlangen data (n=4293). For testing, the best performing methods were applied to remaining data (Leiden n=1000; Erlangen n=478) for an unbiased evaluation.

**Results:**

For the Leiden data set, the word-matching algorithm demonstrated mixed performance (AUROC 0.90; AUPRC 0.33; F1 score 0.55), and 4 methods significantly outperformed word-matching, with support vector machines performing best (AUROC 0.98; AUPRC 0.88; F1 score 0.83). Applying this support vector machine classifier to the test data resulted in a similarly high performance (F1 score 0.81; positive predictive value [PPV] 0.94), and with this method, we could identify 2873 patients with rheumatoid arthritis in less than 7 seconds out of the complete collection of 23,300 patients in the Leiden electronic health record system. For the Erlangen data set, gradient boosting performed best (AUROC 0.94; AUPRC 0.85; F1 score 0.82) in the training set, and applied to the test data, resulted once again in good results (F1 score 0.67; PPV 0.97).

**Conclusions:**

We demonstrate that machine learning methods can extract the records of patients with rheumatoid arthritis from electronic health record data with high precision, allowing research on very large populations for limited costs. Our approach is language and center independent and could be applied to any type of diagnosis. We have developed our pipeline into a universally applicable and easy-to-implement workflow to equip centers with their own high-performing algorithm. This allows the creation of observational studies of unprecedented size covering different countries for low cost from already available data in electronic health record systems.

## Introduction

Electronic health records (EHR) offer an interesting collection of clinical information for observational research, yet a crucial step is an accurate identification of disease cases. This is commonly done by manual chart review or by using standardized billing codes. However, these methods are either labor-intensive or prone to including false positives. Previous studies [1] found that using only standardized billing codes, for example, ≥3 International Classification of Diseases, Ninth Revision (ICD-9) rheumatoid arthritis codes, results in a positive predictive value (PPV) of 56% (95% CI 47%-64%). Using a combination of billing code with a disease-modifying antirheumatic drug code (≥1 ICD-9 rheumatoid arthritis code plus ≥1 disease-modifying antirheumatic drug) results in a PPV of 45% (95% CI 37%-53%). Clinical diagnoses can also be inferred by performing naïve word-matching on format-free text fields. This approach does not take into account the provided context and is thus prone to false positives as well.

Alternatively, query-like algorithms can be used. However, these algorithms require knowledge on the diagnosis of interest, biasing the inclusion of potential study cases. For example, when we want to identify patients with rheumatoid arthritis, we can select people with cyclic citrullinated peptide antibodies that were treated with methotrexate. Those identified likely concern true cases of rheumatoid arthritis but are biased as patients with rheumatoid arthritis do not always receive methotrexate and do not all have cyclic citrullinated peptide–positive tests. On the other hand, selecting only methotrexate would create many false positives as methotrexate is prescribed for many other rheumatic diseases. An additional disadvantage is that rule-based algorithms tend to be center-specific and perform less well in other clinics [2].

Advancements in natural language processing and machine learning have created great potential for processing format-free text data such as those in EHRs [2,3]. A major advantage of machine learning is that it can learn extraction patterns from a set of training examples, relieving the need for extensive domain knowledge. We set out to explore the utility of machine learning methods to identify patients with rheumatoid arthritis from format-free text fields in EHRs. As machine learning methods learn from presented training examples, they can suffer from intercenter variability due to different notation characteristics in EHRs [2].

Therefore, the aim of this study was to develop a broadly applicable workflow that employs machine learning methods to identify patients with rheumatoid arthritis from format-free text fields of EHRs. Additionally, the workflow should be easy to implement and require only the annotation of a subset of the total data set.

## Methods

### Patients’ Data Collection

#### Overview

For this study, we employed 2 data sets: Leiden (the Netherlands) and Erlangen (Germany). See Multimedia Appendix 1 (Table S1) for a convenient overview of the study outline for both centers.

#### Leiden Data Set

**Figure 1:**
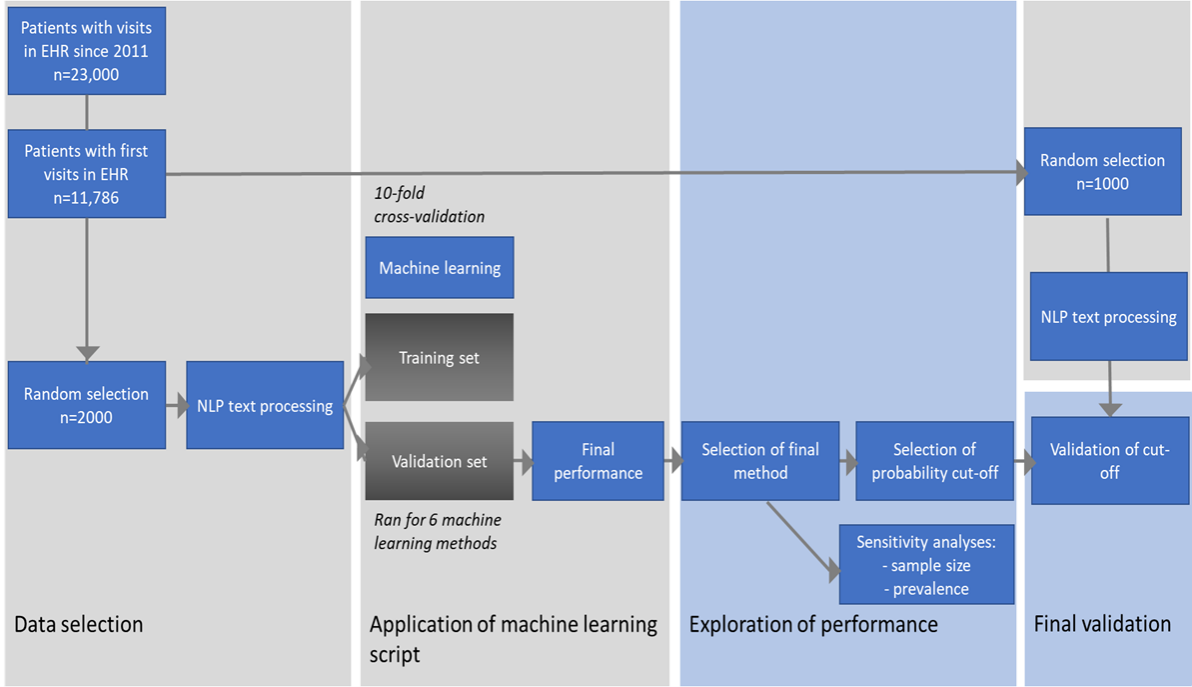
Study outline of the Leiden cohort. EHR: electronic health record; NLP: natural language processing.

We retrieved EHR data from patients (n=23,300) who visited the rheumatology outpatient clinic of the Leiden University Medical Centre since 2011 (Figure 1). We used the *Conclusion* section of the patient records, which consisted of format-free text fields describing the symptoms and (differential) diagnoses of the patient. From these dossiers, 11,786 patients had a first visit after the initiation of the digital system in 2011 [4]. We randomly selected 3000 patients from these newly referred patients and extracted all of their entries for up to 1 year of follow-up. A clinician manually reviewed all entries and annotated the final diagnosis based on all entries. The data were divided into 2 independent sets with a 66/33 split: Leiden-A (n=2000) for model selection, training, and validation and Leiden-B (n=1000) for independent testing. The study was approved by the local ethics board.

#### Erlangen Data Set

After model selection, training, and validation analyses were performed on the Leiden data, we evaluated the universal applicability of our pipeline by applying it to the EHR data from a second center. We retrieved admission notes from the EHR database of University Hospital Erlangen (Department of Internal Medicine 3 Rheumatology and Immunology, Universitätsklinikum). The *course & assessment* component was used because it featured the patient status descriptions. These data consisted of 4771 patients in total featuring all their entries up to 1 year of follow-up. A health care professional manually reviewed all entries and annotated the final diagnosis based on all entries. The Erlangen data set was divided into 2 independent sets with a 90/10 split: Erlangen-A (n=4293) for model and Erlangen-B (n=478) for testing. The study was approved by the local ethics board.

### Training, Model Selection, and Validation (Leiden-A and Erlangen-A)

#### Preprocessing Format-Free Text

We employed spell check and several natural language processing techniques to preprocess the extracted text with scikit-learn tools provided by Pedregosa et al [5]. The pipeline can be divided into 5 steps: word segmentation, lowercase conversion, stop word removal, word normalization, and vectorization. First, we segmented the text into words, splitting by spaces and special characters. Next, we converted the text to lowercase and removed the irrelevant but highly prevalent stop words. Morphological variation was further reduced by applying lemmatization to normalize words to their base form. The tools provide lemmatization tools for many languages; we used the Dutch and German language tools. Segmented words were then aggregated by grouping neighboring words into sets of 3 (ie, *n*-grams such as *patient*, *verdenking artritis*). Finally, a *term frequency by inverse document frequency* transformation, which builds a clinical vocabulary and weighs words according to their occurrence, was applied to vectorize the text data.

#### Training and Machine Learning Model Selection

We tested the following machine learning methods: naïve Bayes [6], neural networks [7], random forest [8], support vector machine [9], gradient boosting [10], decision tree [8], and a random classifier, which assigns class labels at random with frequencies equal to those observed in the training set (parameters are shown in Table S2, Multimedia Appendix 1). Default scikit-learn implementations were used to create the machine learning models [11].

Furthermore, we employed a naïve word-matching algorithm that assigns rheumatoid arthritis status to a sample when the text contained rheumatoid arthritis (in German or Dutch) or its abbreviation appeared in the chart. Each classifier gives a score between 0 and 1 that we interpreted as a probability for each sample to be a case.

We randomly split the Leiden-A and Erlangen-A in train and validation sets using a 10-fold cross-validation procedure for model selection [12]. In short, for each sample set, different models were trained and evaluated in equally sized training and validation sets. Classification performances in the validation sets were then averaged over the samples to give robust estimates of each individually evaluated method to annotate unseen EHR records with a rheumatoid arthritis status.

#### Performance Validation

As each classifier generates a probability score of a rheumatoid arthritis, the performance of a classifier can be tested by applying different cut-offs for case identification. With these probabilities, we first generated receiver operating characteristic curves, plotting the true positive rate against the false positive rate for all probability scores. Second, we created precision-recall curves, plotting the precision (PPV) against the recall (sensitivity or true positive rate) for all score thresholds. Classification performance was then measured using the area under the receiver operating characteristic curve (AUROC) and the area under the precision curve (AUPRC) [11]. For data sets with low case prevalence (imbalanced data), AUROC can be inaccurate and using AUPRC is preferred [13].

To determine whether the performance of the method significantly differed from that of the word-matching method, we implemented the 5×2 cross-validation procedure described by Dietterich [14]. The 5×2 cross-validation procedure splits the data into 2 equal sized sets each repetition. The differences between the classifiers are then estimated with a two-tailed paired *t* test with a significance level of 0.05. This approach takes into account the problem of dependence between the measurements.

The F1 score served as the primary criterion for picking the final method. The F1 score reflects the trade-off between precision and recall as it is the harmonic mean of the two [15]. The best performing model was compared to the other classifiers with two-tailed paired *t* tests (α=.05) in the 5×2 cross-validation, to evaluate whether the best performing model significantly outperformed the other candidates.

#### Sensitivity Analyses

We ran 2 sensitivity analyses on the Leiden data. To evaluate the influence of sample size on the performance of a classifier, we employed the classifier on the Leiden-A data set with decreasing sample sizes within the same 10-fold cross-validation setup. To test the effect of disease prevalence on the classifier’s performance, we created subsets of the Leiden-A set with different fractions of patients with rheumatoid arthritis, applied the classifier to this data and compared the AUPRC between the subsets.

### Final Method Testing of Case Identification (Leiden-B and Erlangen-B)

In the final test phase (using the B data sets), we obtained reliable estimates of the selected model’s performance. We applied the trained model for the best performing method from the A data sets directly to the B data sets (Leiden-B, n=1000; Erlangen-B, n=478). To make a final call on rheumatoid arthritis status, one must define a threshold for the probability. The final test characteristics of the model are affected by the chosen probability cut-off. We report the PPV, sensitivity, and F1 score for each B data set at 2 operator points learned from the A data sets: (1) optimized PPV, thus favoring high-certainty cases and (2) optimized sensitivity, thus favoring the inclusive selection of cases.

### Implementation and Availability

Machine learning methods, model training, and evaluations were performed with the scikit-learn package (version 0.21.2) in Python (version 3.5) [11]. At all times, default implementations and default settings were used. All scripts including instructions on how to apply the methods are posted online [16].

## Results

### Data

Leiden-A (n=2000) and Leiden-B (n=1000) annotated data sets had nearly equal percentages of patients with rheumatoid arthritis (Leiden-A: 154/2000, 7.7%; Leiden-B: 84/1000, 8.4%). Erlangen-A (n=4293) and Erlangen-B (n=478) annotated data sets also had nearly equal percentages of patients with rheumatoid arthritis (Erlangen-A: 1071/4293, 24.9%; Erlangen-B: 112/478, 23.4%).

### Leiden

#### Preprocessing

We found a total of 114,529 words and 8355 unique words in the Leiden-A data after segmentation. With lemmatization and lowercase conversion, the number of unique words was 8141. After removing the most common words with a stop word filter, only 88,524 words and 8078 unique words remained. There were 133,161 unique word combinations (*n*-grams) in the text. The term frequency by inverse document frequency transformation resulted in a sparse matrix of 2000×133,161.

#### Performance Evaluation of Machine Learning Methods

**Figure 2:**
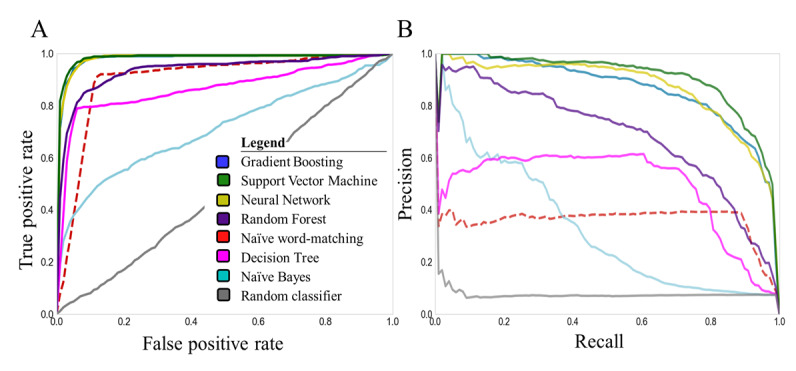
(A) Receiver operating characteristics and (B) precision-recall curves for all machine learning methods (solid lines) and the naïve word-matching method (dotted line) in the training set (Leiden-A).

Naïve word-matching had overall a good performance (AUROC: mean 0.90, SD 0.02), which was significantly better (*P*<.001) than that of a random classifier (AUROC: mean 0.50, SD 0.01). Although naïve word-matching showed good overall test performance, it had a low AUPRC value (mean 0.36 SD 0.07), indicating that the naïve word-matching would generate many false positives. Four machine learning methods outperformed naïve word-matching (AUROC: naïve Bayes mean 0.71, SD 0.03, *P*=.003; neural network: mean 0.98, SD 0, *P*=.005; random forest: mean 0.95, SD 0.01, *P*=.007; support vector machine: 0.98, SD 0.01, *P*=.004; gradient boosting: mean 0.98, SD 0.01, *P*=.003; decision tree: mean 0.86, SD 0.05, *P*=.06) (Figure 2).

The support vector machine had the highest performance in comparison to that of word-matching (AUPRC: mean 0.90, SD 0.02; F1 score: mean 0.83 SD 0.02, *P*<.001). However, the 5×2 cross-validation paired *t* tests revealed that the differences for gradient boosting (*P*=.61), neural network (*P*=.18), and random forest (*P*=.10) were not significant (Multimedia Appendix 2).

#### Sensitivity Analyses

We did not observe any significant loss of precision when lowering the number of training samples from 1000 (original) to 600 patients (Multimedia Appendix 3). Neither the AUROC nor the AUPRC showed a significant difference (*P*=.17 and *P*=.11, respectively). Only when reducing the training set to 450 entries did we observe a significant discrepancy (*P*=.005 and *P*=.005, respectively).

The classifier’s performance maintained an AUPRC >0.80 in settings with highly different disease prevalence (Multimedia Appendix 4). Only when disease prevalence was below 4% or above 50% did we detect a difference in performance compared to that of the initial 8% prevalence.

#### Cut-Off Selection

**Figure 3:**
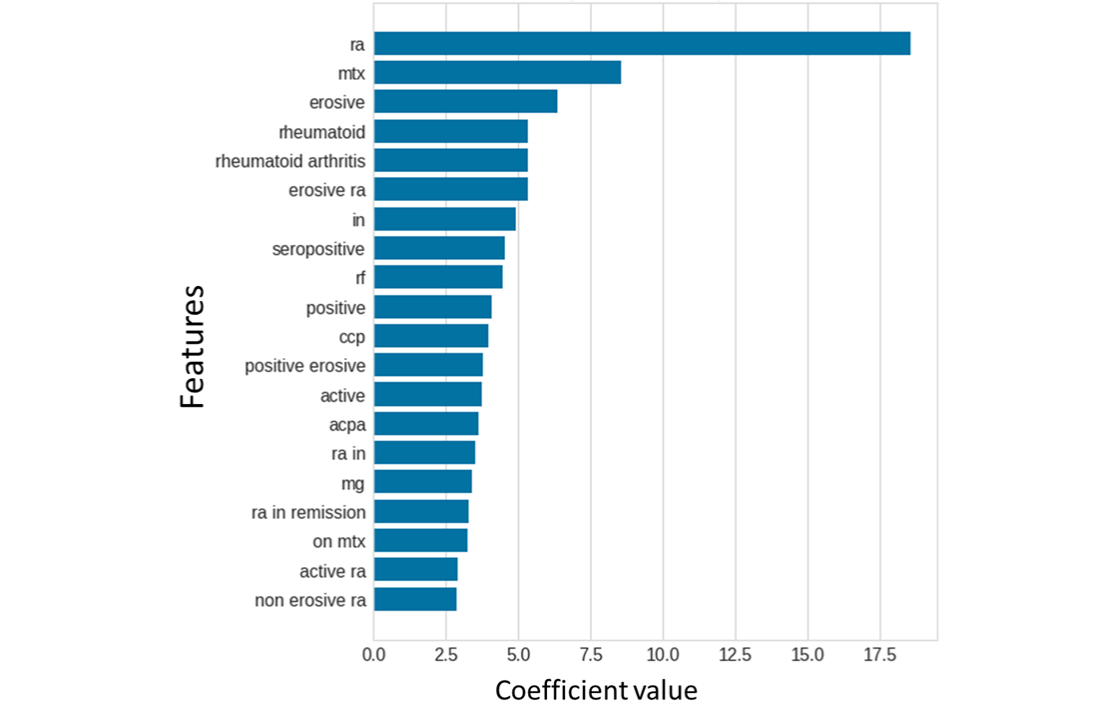
The relative importance (coefficients) of the top 20 features in the Leiden-A data set according to the final support vector machine model. The initial data was in Dutch, we translated the words to English in this figure to improve readability.

**Figure 4:**
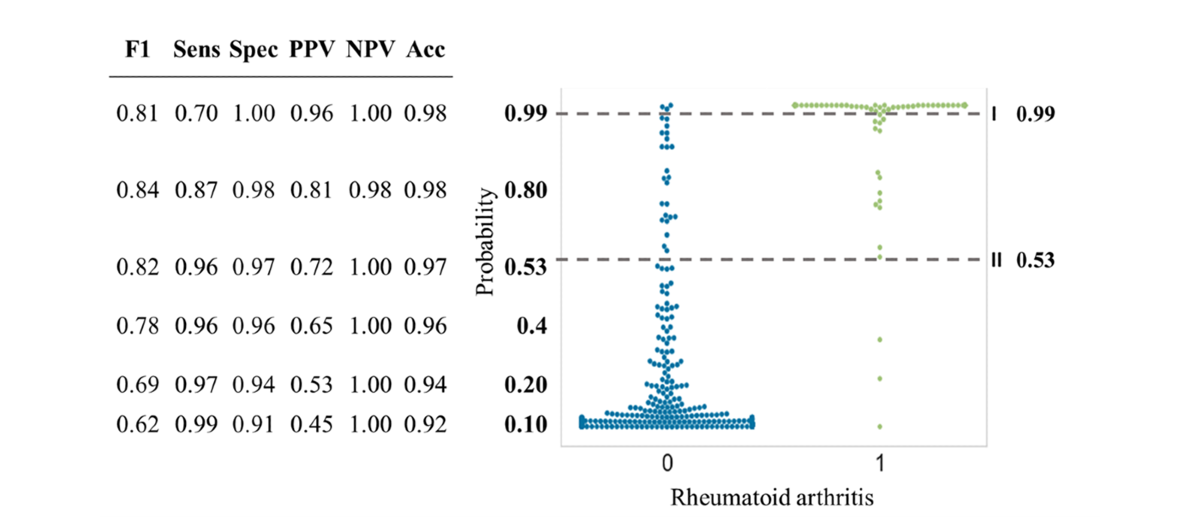
Swarm plot depicting the support vector machine–derived probability of being either non-rheumatoid arthritis (blue) or rheumatoid arthritis (green) for the Leiden-A data set. The dotted lines display the optimal cutoffs. Sens: sensitivity, Spec: specificity; PPV: positive predictive value; NPV: negative predictive value; Acc: accuracy; F1: F1 score.

We picked the support vector machine classifier with the median performance in the training stage. This classifier assigns a probability of being a rheumatoid arthritis to each patient by summing the coefficients of the features present in the clinical notes of the patient (Figure 3). The probability cut-offs for optimized PPV (>0.95) and optimized sensitivity (>0.95) were 0.99 and 0.53, respectively (Figure 4).

The probability cut-off for optimized PPV resulted in the following test characteristics: PPV 0.96, sensitivity 0.70, specificity 1.00, negative predictive value [NPV] 1.00, and F1 score 0.81. The probability cut-off for optimized sensitivity resulted in the following test characteristics: PPV 0.72, sensitivity 0.96, specificity 0.97, NPV 1.00, and F1 score 0.82.

#### Final Method Testing of Case Identification

In the Leiden-B data set, rheumatoid arthritis support vector machine classifier (Table 1) identified 64 cases with a cut-off of 0.99 (with corresponding PPV 0.94, sensitivity 0.71, specificity 1.00, NPV 0.97, and F1 score 0.81) and 104 cases with a cut-off of 0.53 (with corresponding PPV 0.75, sensitivity 0.93, specificity 0.97, NPV 0.99, and F1 score 0.83). In the complete Leiden data set of 23,300 patients using the first (precise) cut-off resulted in 2873 cases of rheumatoid arthritis and the second (inclusive) cut-off resulted in 6453 cases of rheumatoid arthritis.

**Table 1:** Support vector machine confusion matrices for the Leiden-B test set (n=1000) .

Clinician-based	Support vector machine 1 (cut-off=0.99)		Support vector machine 2 (cut-off=0.53)	
	Non–rheumatoid arthritis	Rheumatoid arthritis	Non–rheumatoid arthritis	Rheumatoid arthritis
Non–rheumatoid arthritis	912 (true negative)	4 (false positive)	890 (true negative)	26 (false positive)
Rheumatoid arthritis	24 (false negative)	60 (true positive)	6 (false negative)	78 (true positive)

### Validation of Workflow in Erlangen Data

#### Training and Model Selection

**Figure 5:**
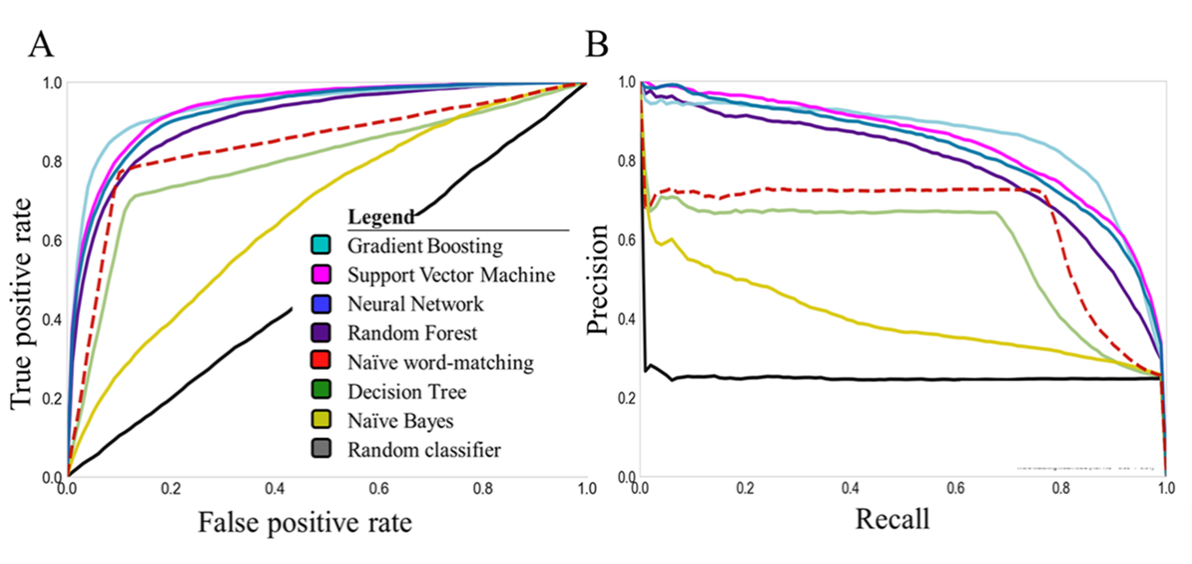
(A) Receiver operating characteristics and (B) precision-recall curves for all machine learning methods (solid lines) and the naïve word-matching method (dotted line) in the training set (Erlangen-A).

To evaluate the universal applicability of the workflow, we employed the full pipeline on Erlangen data sets. Again, we ran all machine learning methods to find the best performing method using the Erlangen-A data set. Gradient boosting achieved the best performance (AUROC 0.94; AUPRC 0.85; F1 score 0.81) (Figure 5). The probability cut-offs for optimized PPV (> 0.90) and optimized sensitivity (>0.90) were 0.79 and 0.19, respectively (Multimedia Appendix 5).

#### Final Method Testing of Case Identification

When we applied the model on the test data set (Erlangen-B), we obtained similar performance (Table 2) with the predefined cut-offs as those found for the training data set (Erlangen-A). The gradient boosting classifier identified 59 cases with a cut-off of 0.79 (with corresponding PPV 0.97, sensitivity 0.51, specificity 0.99, NPV 0.87, and F1 score 0.67) and 131 cases with the cut-off of 0.19 (with corresponding PPV 0.72, sensitivity 0.84, specificity 0.90, NPV 0.95, and F1 score 0.77).

**Table 2:** Gradient boosting confusion matrices for the Erlangen-B test set (n=478).

Clinician-based	Gradient boosting 1 (cut-off=0.79)		Gradient boosting 2 (cut-off=0.19)	
	Non–rheumatoid arthritis	Rheumatoid arthritis	Non–rheumatoid arthritis	Rheumatoid arthritis
Non–rheumatoid arthritis	364 (true negative)	2 (false positive)	329 (true negative)	37 (false positive)
Rheumatoid arthritis	55 (false negative)	57 (true positive)	18 (false negative)	94 (true positive)

## Discussion

### Principal Findings

Our study describes the results of a pipeline that applies multiple machine learning methods as well as naïve word-matching to create algorithms of case selection (patients with rheumatoid arthritis in our example) from electronical medical records. We observed that most methods outperform a naïve word matching algorithm. Our pipeline created algorithms on both Dutch and German data that showed a high performance in the testing and validation phase (F1 score 0.83 and 0.82 respectively). When we defined the cut-offs for case selection from the first data set aiming for either a high sensitivity or high PPV, we observed that the performances were robust in the second data sets (Leiden-B: PPV 0.94 and sensitivity 0.93; Erlangen-B: PPV 0.97 and sensitivity 0.84).

We believe that our approach of making a center-specific algorithm is more attractive than the application of an algorithm developed elsewhere, since our method is more precise, doesn’t require standardization, and most importantly, it ensures high performance within the center. Our method only requires similar effort as the application of predefined algorithms, namely chart reviewing a subset of data. Furthermore, our workflow respects the user’s requirements regarding the case selection. The case selection can be tailored to being highly precise or sensitive depending on the chosen cut-off.

Furthermore, this study shows the power of machine learning approaches to generate cohorts of patients in seconds, laying a foundation for allowing studies of cohorts with an unprecedented low cost.

When applying our support vector machine classifier on the complete Leiden University Medical Centre’s database of 23,300 cases (including the 3000 annotated records) we identified 2873 rheumatoid arthritis cases when employing the stringent probability threshold of 0.99. The automatic annotation only took 6.17 seconds, a fraction of the amount of time it would take to review the medical charts manually.

### Future Directions

Our aim was to implement a broadly applicable workflow. The current versions require installing Anaconda (version 5.1.0) and Python (version 3.6). Researchers without any computational experience might feel certain reluctance to start the pipeline. We tested (without quantification) how easy someone outside our center could run the pipeline, by sending the scripts to scientists at Erlangen. Though they implemented the pipeline with relative ease, we do acknowledge that it was done by someone with experience in computational languages. Also, testing the pipeline in Erlangen exposed some unclarities in the scripts, which have been improved. The next step would be to perform a usability study, where we could ask users for their experience as well as test how much time it takes them to get the script running. We could further improve the usability of the pipeline by creation of a web-based interface where people could upload their data and get back their results automatically. This would require substantial computational resources as the data sets are large. In addition, we would need to ensure encryptions processes as clinical notes have a high risk to breach privacy.

### Limitations

We want to note 3 important shortcomings of our study. The first limitation is that deploying the pipeline requires user familiarity with implementation software. Our proposed workflow facilitates building a classifier with a step-by-step implementation. Affinity with programming is not required, because all functions for training and evaluation are already provided. However, some software experience is beneficial when setting up the environment for the pipeline to run. With the emergence of machine learning and natural language processing we would argue that it becomes increasingly useful to possess the skills required to implement software.

Second, we acknowledge that the workflow was evaluated in only 2 centers, both with Germanic languages. Although the pipeline provides language-specific preprocessing with pretrained tools for most languages, it would be interesting to investigate if similar performance can be achieved in centers with low lexical similarities to the Dutch language (eg, languages without a Latin-based alphabet).

Finally, we acknowledge that the models’ performances can be further optimized by fine-tuning hyperparameters. These are parameters of the machine learning method that are provided prior to training the machine learning method. Additionally, it is possible to adjust the size of the n-grams to improve the performance. Since our models consistently performed very well in training and testing, we did not optimize any parameters in our study. Furthermore, we only evaluated a handful of candidate machine learning methods. Our selection is by no means an exhaustive list of available techniques in the field. We selected these methods as they cover a variety of machine learning method and are widely known.

### Lessons Learned

We were able to conduct a stringent flow of training and testing, whereby we used several independent data sets to, first, optimize the classifiers, and second, to ensure reliable calculations of the classifiers’ performances by using k-fold cross-validation and both receiver operating characteristic and precision recall curves on 10-fold cross-validation, providing a good indication of performance on unseen data.

To select the best classifier, we performed paired *t* tests on 5×2 cross-validation rather than 10-fold cross-validation. Although performing a paired *t* test on 10-fold cross-validation is a very common practice, we learned that this test is not recommended. The correlation between overlaps violates the *t* test’s assumption of independence, resulting in more false positives (increased type I error); 5×2 cross-validation splits the data set 50/50 and is, therefore, more suitable for statistical analysis. However, 5×2 cross-validation is confined to a small training set, which is why we also used 10 cross-validations to approximate the performance on unseen data.

Our study is not the first to examine methods for disease identification from EHR [3]. Studies have employed high-throughput methods on structured data such as ICD (billing) codes. Regrettably, such codes have a poor performance because they describe why a patient is examined, which does not strictly mean that a patient has that diagnosis. More successful algorithms (often called phenotype algorithms) combined a variety of methods including rule-based case identification and natural language processing [2]. Though these algorithms have a good median performance when tested in multiple clinics, on an individual center PPV varies (below 0.5 for several clinics) [2]. Moreover, several centers required additional tailoring to allow application of the algorithms. This is not surprising since health clinics have different protocols for registering information.

As gold standard, we purposely chose the diagnosis of the treating rheumatologist in contrast to counting the disease classification criteria [17,18]. The problem with the latter is that classification criteria have been developed for research and not for clinical practice where all information including additional tests in the differential diagnostic workup are taken into account. Moreover, the exact information for individual criteria is often not precisely registered in EHRs.

We ran several sensitivity analyses to explore the influence of disease prevalence and number of selected patients on the model's performance. The support vector machine classifier was robust over different selections of training data (low standard error on the cross-validation results), number of training samples, and imbalances of case number. These analyses also showed that in our Leiden data the annotation of 600 patients would have been sufficient to build a reliable classifier. We acknowledge that due to difference in feature variance, the optimal number of patients required to train the classifier might differ between centers.

### Generalizability of the Workflow

The support vector machine was the best classifier for Leiden-A (F1 score 0.83), although the difference was not significant with respect to the gradient boosting, neural networks, and random forest. The support vector machine was employed in the independent Leiden-B data set with similarly good performance (F1 score 0.81). We predefined 2 thresholds of the rheumatoid arthritis support vector machine probabilities on the first Leiden data (Leiden-A) aiming for either a high precision (PPV 0.94), or a high sensitivity (sensitivity 0.93). When we applied these predefined cut-offs in the second set of patients we obtained similarly high test characteristics (PPV 0.96, sensitivity 0.70, specificity 1.00, NPV 1.00 with the highly precise threshold, and PPV 0.72, sensitivity 0.96, specificity 0.97, NPV 1.00 with the highly sensitive threshold). Finally, we ran the same workflow of training and testing as employed on the Dutch Leiden data to the German Erlangen data. Again, we built a high performing classifier (in this case gradient boosting performed best) that gave consistent results for both settings (PPV 0.97, sensitivity 0.51, specificity 0.99, NPV 0.87 with the highly precise threshold, and PPV 0.72, sensitivity 0.84, specificity 0.90, NPV 0.95 with the highly inclusive threshold).

The gradient boosting has the best performance in the Erlangen data, while in the Leiden data the support vector machine performs the best. This is not necessarily surprising, as “there is no such thing as a free lunch” (meaning that a universal best algorithm does not exist) [19]. The high performance of the support vector machine is achieved by generalizing the Leiden data. There is no guarantee that the technique used in the Leiden data set will also perform the best in the Erlangen data set. Notably, in each data set, both methods performed very well with only very modest differences. The slight deviations in performance between the methods could be caused by language differences and characteristic notations of the center.

**Figure 6:**
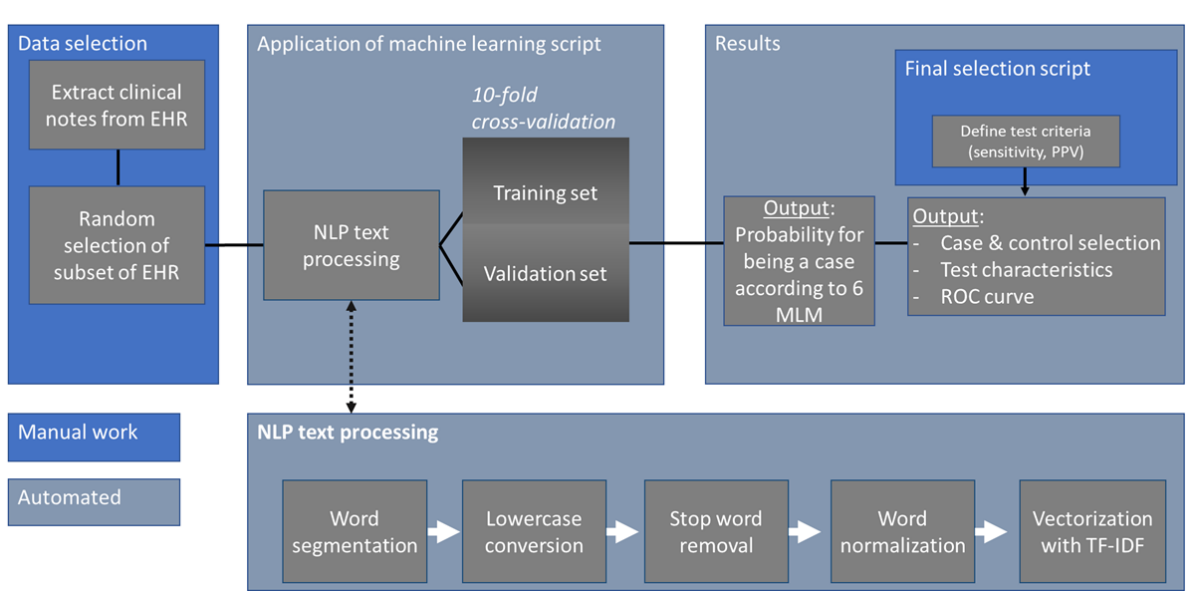
Flowchart describing the steps to apply the machine learning scripts to new data. EHR: electronic health record; MLM: machine learning method; NLP: natural language processing; PPV: positive predictive value; ROC: receiver operating characteristic; TF-IDF: term frequency by inverse document frequency.

In accordance with the FAIR principles [20], we have made all our scripts publicly available and optimized them so scientists may use them regardless of prior experience (Figure 6) [16]. We advise centers not to use our specific classifier but to follow the workflow as presented in this paper and build a classifier that fits the local data best.

### Conclusion

The workflow facilitates the production of highly reliable center-specific machine learning methods for the identification of patients with rheumatoid arthritis from format-free text fields. Our results suggest that our workflow can easily be applied to other EHRs or other diseases and is not restrained by specific language, EHR software, or treatments. This methodology of machine learning for EHR data extraction facilitates cohort studies (with regard to cost and size).

## Appendix

Multimedia Appendix 1 Overview of study details.

Multimedia Appendix 2 Average F1 score for all machine learning methods (Leiden-A).

Multimedia Appendix 3 Performance of SVM on increasing training set within the Leiden-A data set.

Multimedia Appendix 4 Performance of SVM on increasing prevalence in Leiden-A.

Multimedia Appendix 5 Swarm plot depicting gradient boosting–derived probability of being rheumatoid arthritis.

## Abbreviations

AUPRC: area under the precision recall curve
AUROC: area under the receiver operating characteristic curve
EHR: electronic health record
ICD-9: International Classification of Diseases, Ninth Revision
NPV: negative predictive value
PPV: positive predictive value
