# Supplementary material for: Machine Learning Electronic Health Record Identification of Patients with Rheumatoid Arthritis: Algorithm Pipeline Development and Validation Study
Source: JMIR Med Inform. 2020 Nov 30;8(11):e23930. doi: 10.2196/23930 (PMC7735897; doi:10.2196/23930)
Supplement: Multimedia Appendix 1 [file medinform_v8i11e23930_app1.docx]

# Multimedia Appendix

**Supplementary Table 1** Overview of study outline with corresponding results.

| **Data** | **Analyses** |
| --- | --- |
| **Leiden** |  |
| Leiden-A  N = 2,000 | Comparison of MLMs with naïve word matching to build the optimal algorithm (Fig. 2)  Selection of final algorithm and definition of optimal cut-off (Fig. 4)  Sensitivity analyses   - Explore effect of sample size on model’s performance (Multimedia Appendix 3) - Explore effect of case prevalence on model’s performance (Multimedia Appendix 4) |
| Leiden-B  N = 1,000 | Test performance of developed model (Table 1) |
| **Erlangen** |  |
| Erlangen-A  N = 4,293 | Comparison of MLMs with naïve word matching to build the optimal algorithm (Fig. 5)  Selection of final algorithm and definition of optimal cut-off (Multimedia Appendix 5) |
| Erlangen-B  N = 478 | Test performance of developed model (Table 2) |

**Supplementary Table 2** Hyperparameters used by the default sci-kit learn [5] implementations of the classifiers.

| Method | Parameter 1 | Parameter 2 | Parameter 3 |
| --- | --- | --- | --- |
|  | **C** | **Kernel** |  |
| SVM[9] | 1.0 | Linear |  |
|  | **Criterion** | **N-estimators** |  |
| Random Forest[8] | Gini | 100 |  |
|  | **Criterion** |  |  |
| Decision Tree[8] | Gini |  |  |
|  | **Solver** | **Hidden layers** | **Activation** |
| Neural Networks[7] | L-bfgs | 1 (100 units) | Relu |
|  | **Probability density** |  |  |
| Naive Bayes[6] | Multinomial |  |  |
|  | **Criterion** | **N-estimators** | **Loss** |
| Gradient Boosting[10] | Friedman_mse | 100 | Deviance |
