# Supplementary figures and images for: Machine Learning Electronic Health Record Identification of Patients with Rheumatoid Arthritis: Algorithm Pipeline Development and Validation Study
Source: JMIR Med Inform. 2020 Nov 30;8(11):e23930. doi: 10.2196/23930 (PMC7735897; doi:10.2196/23930)

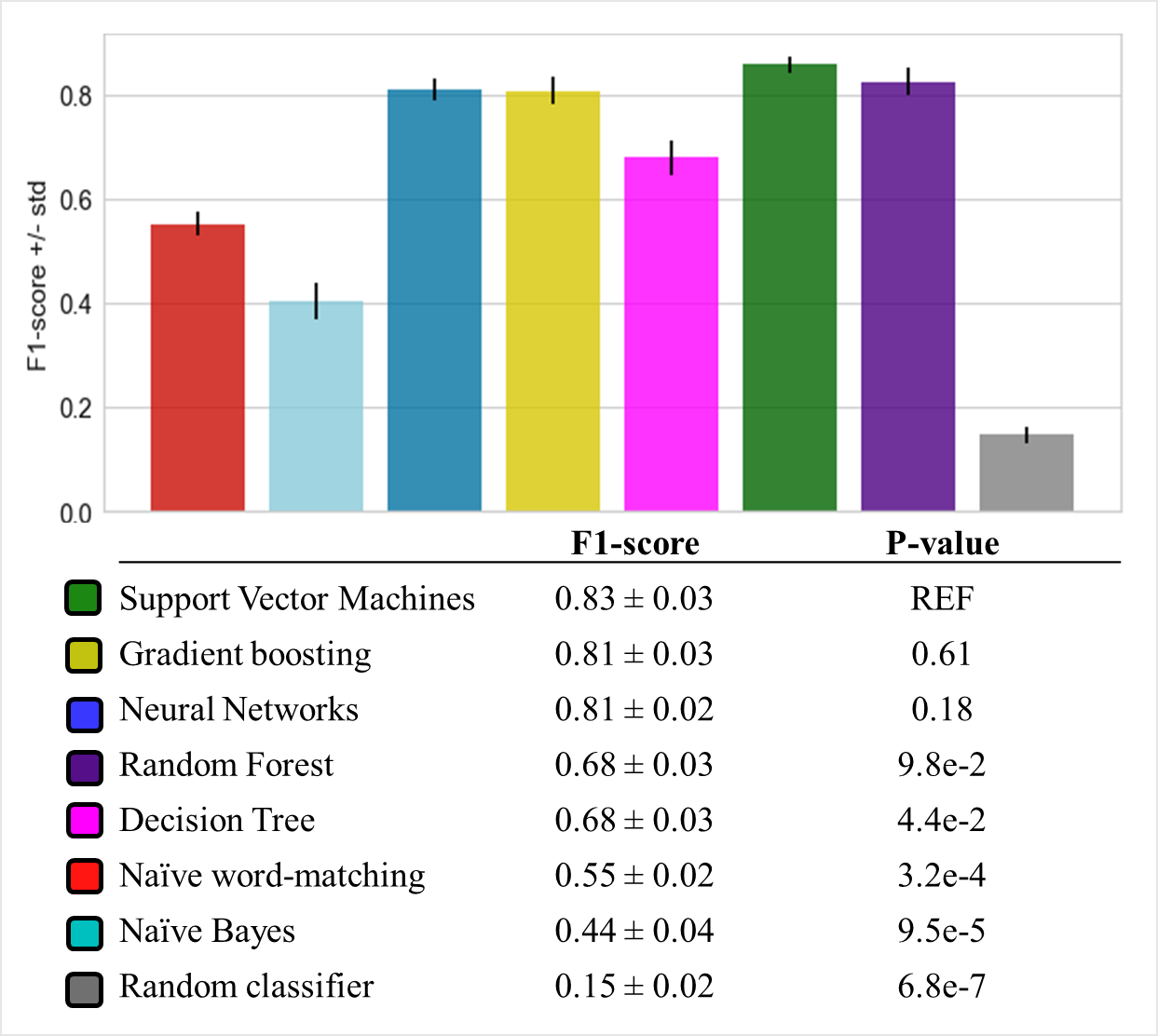

Supplement: Multimedia Appendix 2 [file medinform_v8i11e23930_app2.png]

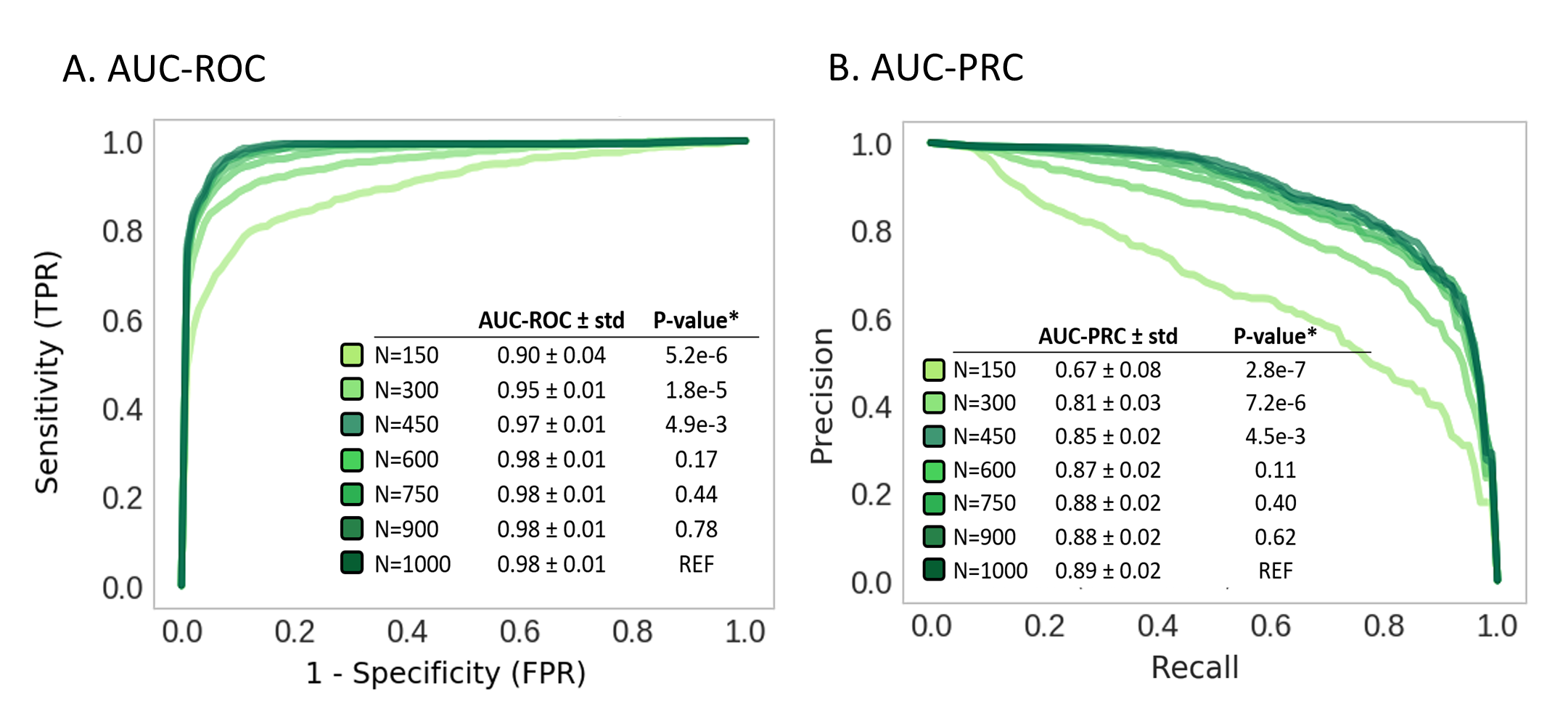

Supplement: Multimedia Appendix 3 [file medinform_v8i11e23930_app3.png]

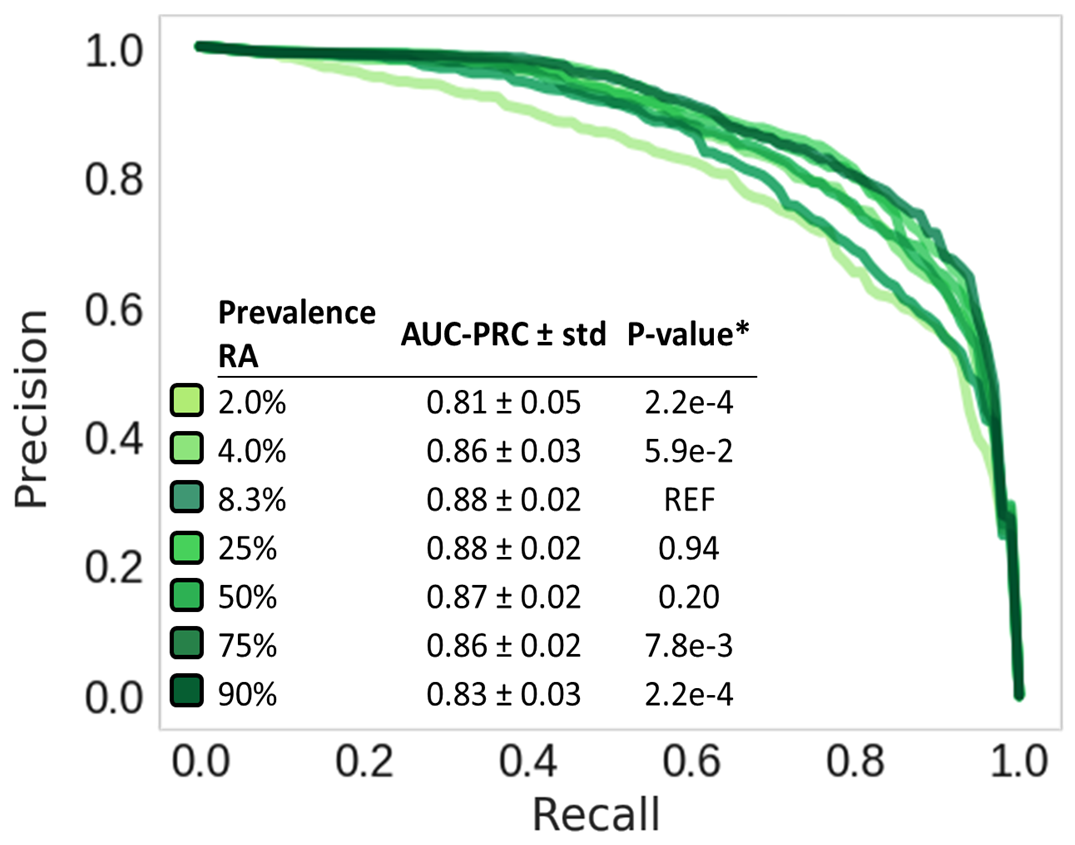

Supplement: Multimedia Appendix 4 [file medinform_v8i11e23930_app4.png]

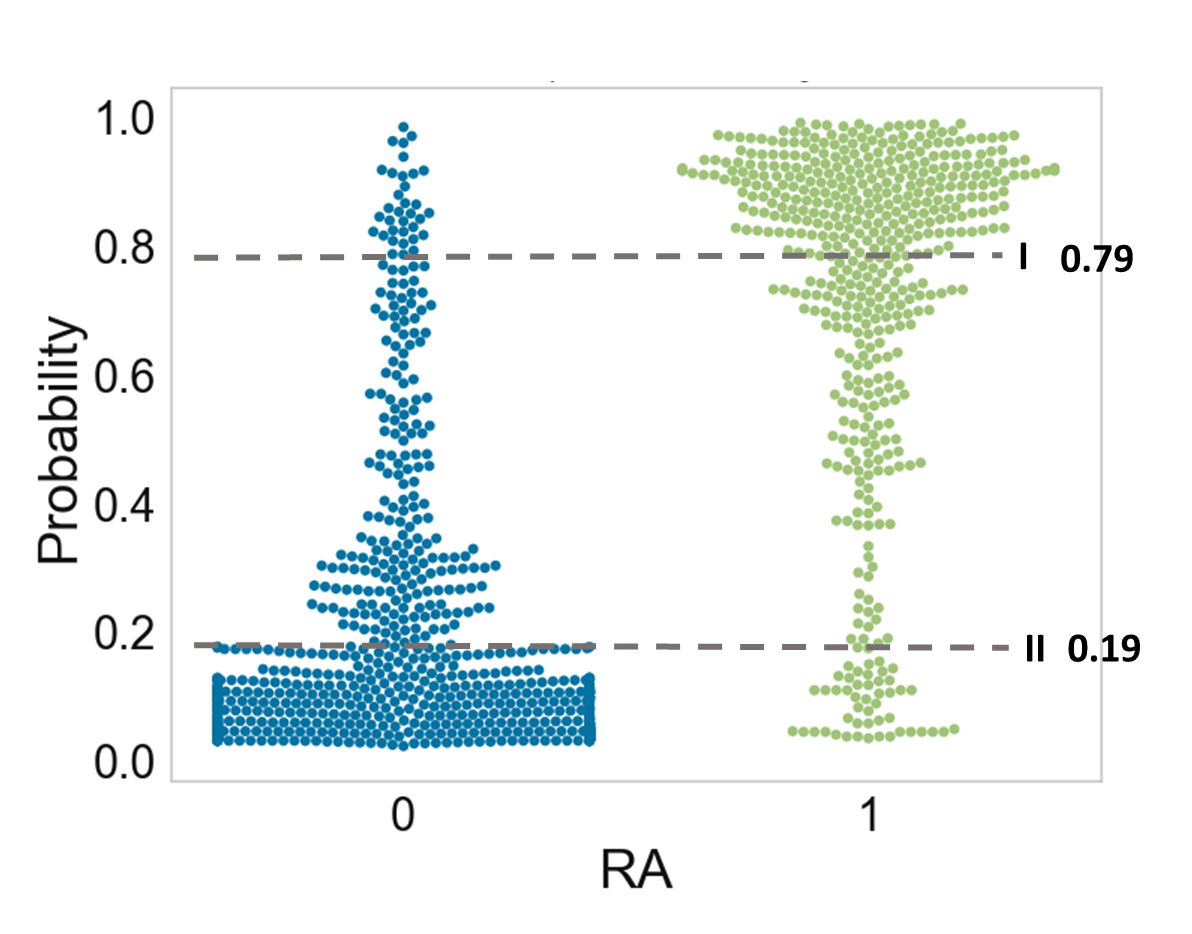

Supplement: Multimedia Appendix 5 [file medinform_v8i11e23930_app5.png]
